# Assessment of the posterior pelvic ring during examination under anesthesia changes surgeons’ classification and treatment decisions of anterior posterior compression pelvic ring injuries

**DOI:** 10.1007/s00590-026-04930-8

**Published:** 2026-09-28

**Authors:** Camryn C. Therrien, Kaj ten Duis, Alessandro Aprato, Hester Banierink, Umberto M. R. Mezzadri, Henry C. Sagi, Jean-Paul P. M. de Vries, Inge H. F. Reininga, Frank F. A. IJpma, Society of European Pelvic and Acetabulum Surgeons

**Affiliations:** 1 Department of Trauma Surgery, University of Groningen, University Medical Center Groningen, Groningen, Netherlands; 2 Department of Orthopedics, University of Turin, Turin, Italy; 3 Department of Orthopedics and Trauma Surgery, ASST GOM Niguarda, Milan, Italy; 4 Department of Orthopedic Surgery and Sports Medicine, University of Cincinnati College of Medicine, Cincinnati, USA; 5 Department of Surgery, University of Groningen, University Medical Center Groningen, Groningen, Netherlands

**Keywords:** Anterior-posterior compression injury, Examination under anesthesia, Pelvic stability, Pelvic fixation

## Abstract

**Purpose:**

Traditionally, examination under anesthesia (EUA) focuses on displacement of the anterior pelvic ring and may fail to detect or localize sacroiliac (SI) joint diastasis in patients with anterior-posterior compression (APC) pelvic injuries. This study evaluates the effect of directly observing sacroiliac (SI) diastasis during EUA, compared to observing only the anterior pelvic ring (AP EUA), on changes in classification and treatment plan.

**Methods:**

Four hundred international pelvic surgeons were invited to participate in an online survey, with one month between two surveys. All surveys included the same 10 cases of APC injuries. One part included AP EUA images, and the other included additional SI EUA images. Questions regarding classification and treatment were asked, including the need for anterior and posterior fixation, and the side of SI joint fixation. The change in management after the addition of SI EUA images was assessed.

**Results:**

One hundred six pelvic trauma surgeons completed the survey with AP EUA images, and 99 also completed the survey with SI EUA images. The addition of SI EUA imaging resulted in a 37% change in classification, with APC1 decreasing from 17% with AP EUA to 10% with SI EUA, APC2 remaining 67%, and APC3 increasing from 17 to 23%. Treatment decisions changed in 29% of all cases. There was an increase in the combined anterior and posterior fixation, from 56 to 74%. Improved lateralization (choice of right or left SI joint fixation) was seen in 4 cases.

**Conclusion:**

In this survey, the addition of SI EUA images according to the modified protocol changed surgeons’ perception of pelvic ring instability and the subsequent treatment strategy chosen. SI EUA images provide surgeons with information that prompts the addition of an SI joint fixation, resulting in an 18% increase in the use of combined anterior and posterior fixation. SI EUA images also improved injury localization, helping surgeons identify which sacroiliac joint required stabilization.

**Supplementary Information:**

The online version contains supplementary material available at https://doi.org/10.1007/s00590-026-04930-8.

## Introduction

 Examination under anesthesia (EUA) is a dynamic fluoroscopic imaging technique used to detect occult instability in patients with anterior–posterior compression (APC) pelvic ring injuries [1]. Because APC injuries are primarily ligamentous in nature [2–4], the degree of instability may be underestimated on static imaging such as radiographs and CT scans, especially when influenced by factors such as patient positioning and the use of pelvic binders [1, 5–9]. It has been demonstrated that EUA can reveal occult instability not apparent on initial static imaging [1, 5, 7, 10, 11]. Moreover, an international survey study showed that the use of EUA according to the protocol by Sagi et al. [1] changes surgeons’ interpretation of injury severity and the resulting treatment choice [12]. The addition of EUA is important as misinterpretation of instability may result in inappropriate management or insufficient fixation, potentially leading to fixation failure, loss of reduction, and poor functional outcomes [1, 5, 13–17].

The original EUA protocol (e.g., AP EUA, focused on anterior pelvic displacement) described by Sagi et al. [1] has been widely adopted. This original protocol relies on cut-off values of anterior pelvic ring diastasis to infer the degree of posterior pelvic ring instability. Specifically, a pubic symphysis diastasis greater than 2.5 cm is considered indicative of anterior sacroiliac (SI) ligament disruption, and rotational instability exceeding 1.0 cm is interpreted as complete disruption of anterior and, to some extent, posterior SI ligaments. In the protocol, treatment recommendations are based on these cut-off values [1]. These cut-off values, however, have not been validated. Cadaveric investigations by Doro et al. were unable to confirm the 2.5 cm cut-off value’s validity in distinguishing the presence of anterior SI ligament disruption, demonstrating that symphyseal diastasis can range from 1.8 to 4.4 cm before anterior SI ligaments fail [18]. To address this limitation, a modified EUA protocol (e.g., SI EUA), incorporating direct fluoroscopic assessment of the sacroiliac joints, was recently introduced and applied to a patient cohort [10]. With the application of this modified protocol, it was observed that a third of patients had posterior pelvic ring instability that would have been overlooked if only the measurements of the anterior pelvis were used [10]. These findings suggest that an assessment solely based on anterior ring diastasis may inadequately determine posterior instability.

**Fig. 1:**
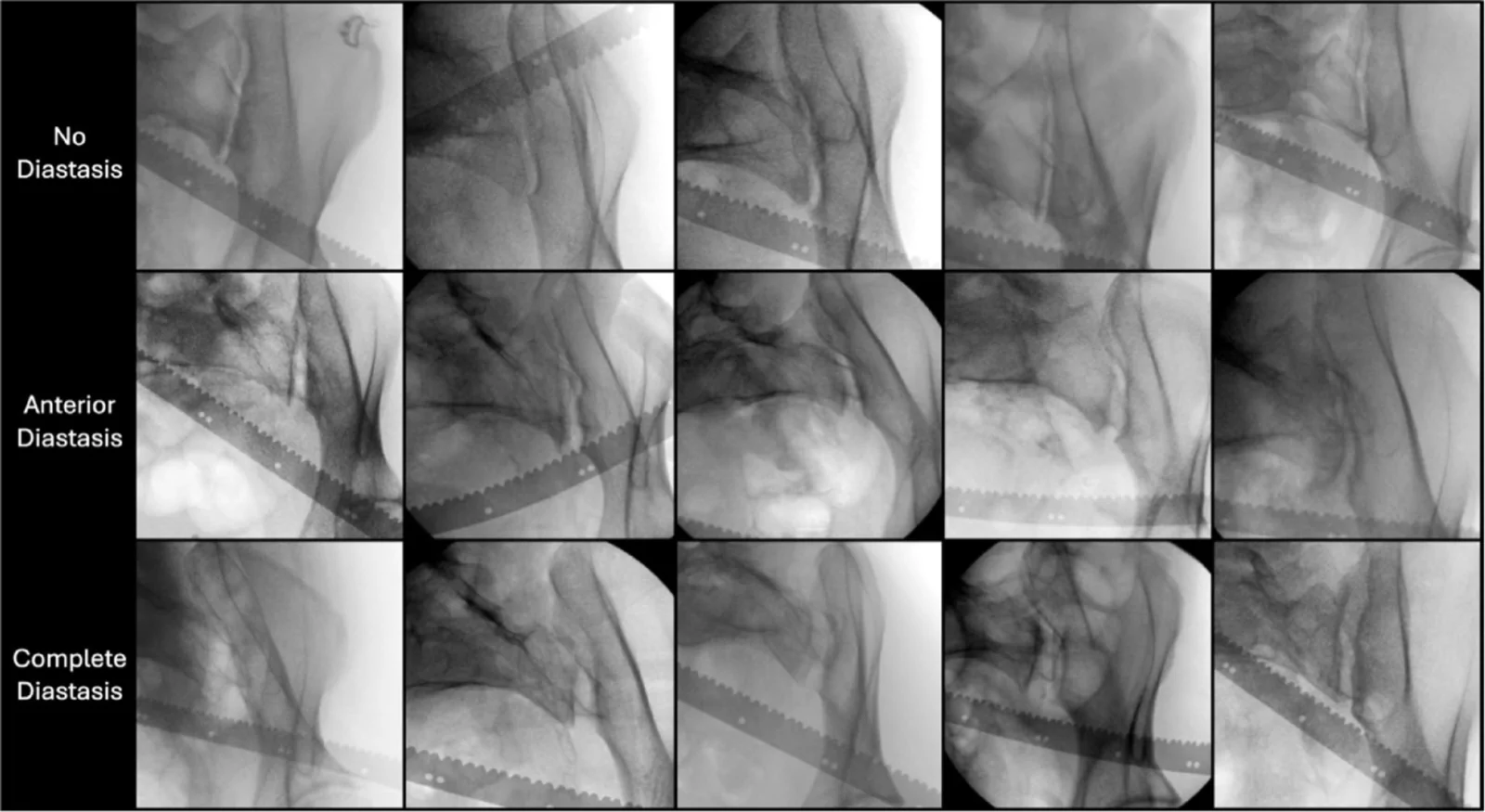
SI EUA images of patients with APC injuries taken in the obturator inlet view during external rotation stress maneuver [10]

The modified SI EUA protocol includes 11 fluoroscopic images evaluating both the anterior and posterior pelvic ring directly. The anterior ring is assessed using five anteroposterior (AP) projections obtained during static, internal rotation stress, external rotation stress, and longitudinal push/pull stress maneuvers, with focus on the pubic symphysis. Posterior ring assessment is performed using static, internal rotation, and external rotation stress maneuvers in obturator inlet projections to directly visualize both SI joints. Figure 1 presents examples of sacroiliac (SI) joints in the obturator inlet view during the external rotation stress maneuver of patients with APC injuries. This technique has been shown to enable the detection of SI joint diastasis not visible on CT imaging, and to improve the localization of posterior instability to inform whether the right or left SI joint should be fixated and identify instability that would have remained undetected if only the anterior ring had been evaluated [10].

Despite these potential advantages, it remains unknown whether incorporating additional SI joint images influences surgeons’ interpretation of APC injury severity or alters subsequent treatment decisions.

To address this, an international survey regarding cases of APC injuries completed by pelvic surgeons was conducted to evaluate the following research questions:

1. Does the addition of SI joint EUA images according to the modified protocol change surgeons’ classification of APC pelvic ring injuries compared to anterior-only AP EUA images?
2. Does the addition of SI joint EUA images according to the modified protocol influence surgeons’ treatment decisions in patients with APC pelvic ring injuries compared to AP EUA images only?

## Methods

A three-part international survey was conducted to evaluate surgeons’ classification and treatment decisions for APC pelvic ring injuries. The same 10 cases were presented in all three parts of the survey. Parts one and two of the survey investigated the change in classification and treatment decisions by surgeons after the addition of AP EUA imaging, compared to initial static radiographic (Antero-Posterior (AP), inlet and outlet projections) and CT imaging only [12].

One month following the completion of the second survey, a third survey was sent, which included the addition of SI EUA images according to the modified EUA protocol [10]. The current study examines the change in classification and treatment decisions by surgeons after the addition of SI EUA images, compared to AP EUA imaging, according to the original protocol by Sagi et al. [1].

The local Medical Ethical Review Board reviewed the methods employed and waived further need for approval (METc 2017/543).

### Case description

The same ten cases were used in all parts of the survey. The case selection criteria were adult patients who had an APC injury [2], based on assessment of the preoperative static radiographic and CT imaging, all of whom underwent EUA with fluoroscopy. To have a more accurate representation of true clinical practice, five of the ten cases were initially managed with pelvic binders during pre-operative CT imaging (binder closed and tightened), and five were managed without a binder [19]. All ten cases had static AP radiographs taken without pelvic binders in place.

### Surveys

Four hundred pelvic surgeons from a variety of countries and hospitals were invited to participate in this online survey via email in March of 2025 [12]. The REDCap (Research Electronic Data Capture) data collection platform was used for the initial invitation and the distribution of all surveys throughout this study. REDCap is a secure, web-based software platform designed to support data capture for research studies [20, 21]. The first survey included questions regarding the surgeon’s individual experience with pelvic surgery and clinical practice.

The survey that included AP EUA images (e.g. focused only on anterior pelvic ring displacement) incorporated static AP radiograph and an axial CT scan of each patient, with the addition of the following four AP EUA images: internal rotation stress (lateral compression), external rotation stress (frog-leg position), push right leg/ pull left leg, and push left leg/ pull right leg [1]. Rather than including the full series of 15 EUA images, we limited the number of dynamic views to four representative stress maneuvers to enhance clarity and reproducibility of interpretation for respondents while maintaining the clinically relevant information needed to assess pelvic ring instability. Each EUA image included a radiopaque ruler, allowing surgeons to take measurements if desired. The same set of questions was asked for all cases (online Appendix 1).

The survey that included SI EUA images comprised the same ten cases presented in a different order than the AP EUA survey. The order of case presentation was the same for all surgeons. This survey was sent one month following completion of the prior survey. This survey included the same static images as in the AP EUA survey, with the addition of one SI image per SI joint with an obturator inlet view for each patient. These images were taken in the external rotation stress (frog-leg position) as described in the modified EUA protocol [10]. It was chosen to only include one image of each SI joint to increase comprehensibility for participants, and the external rotation stress image provides the crucial information needed for assessing the stability of the SI joint. The same questions were asked again for all cases. Specifically, the classification of the injury (e.g., APC1, APC2, or APC3) and the treatment approach (e.g., non-operative, anterior fixation, and right and left SI joint fixation). All responses were included in the analysis, even if the surgeon did not complete all parts of the survey.

### Statistical analysis

For Gaussian distributed data, means and standard deviations (SD) were presented, and for non-Gaussian distributed data, medians and interquartile ranges (IQR) were used. Frequency was presented as n (% of all patients). The statistical analysis was performed using IBM SPSS Statistics (Version 28).

Surgeon information of those who completed each survey was analyzed to assess potential differences between respondents across the two surveys. Non-Gaussian distributed continuous variables were analyzed using the Mann-Whitney U test, and categorical variables were compared using the chi-square test or Fisher’s exact test.

The following treatment groups were made: non-operative, anterior-only fixation, and both anterior and posterior fixation, based on the specific treatment decisions made by the surgeons. If the surgeon’s response did not fit within one of these three groups, it was recorded as ‘other’.

Whether the surgeon’s classification or treatment decision differed on the SI EUA survey compared to the AP EUA was recorded as a changed classification or treatment decision. The number of changed classifications and treatment decisions is presented for each case and in total across the ten cases. Furthermore, all the classifications and treatment decisions from both surveys are presented per case.

An analysis of whether the right or left SI joint was fixated was performed, comparing treatment decisions based on AP EUA images and SI EUA images.

## Results

### Surgeons

One hundred six surgeons completed the survey on AP EUA images, and of those, 99 also completed the survey on SI EUA images one month later. Information regarding the experience and standard practice of surgeons who completed both surveys is presented in Table 1. There were no significant differences in surgeon information between surgeons who completed the two surveys.

**Table 1:** Surgeon information

	AP EUA survey (*n* = 106)	SI EUA survey (*n* = 99)	*p*-value
Specialty			0.958
Trauma surgery, n (%)	79 (76)	72 (73)	
Orthopedic surgery, n (%)	21 (20)	21 (21)	
Other*, n (%)	6 (6)	6 (6)	
Level of trauma center			0.994
Level 1, n (%)	90 (85)	84 (85)	
Level 2, n (%)	12 (11)	11 (11)	
Level 3, n (%)	4 (4)	4 (4)	
Years practicing specialty, median (IQR)	14 (6–20)	14 (6–20)	0.934
Years treating pelvic ring injuries, median (IQR)	10 (5–15)	10 (5–15)	0.912
Pelvic injuries treated per year, median (IQR)	50 (20 −70)	50 (20 − 70)	0.885
Uses EUA in their standard practice, n (%)	53 (50)	51 (52)	0.828

### Changes in classification

**Fig. 2:**
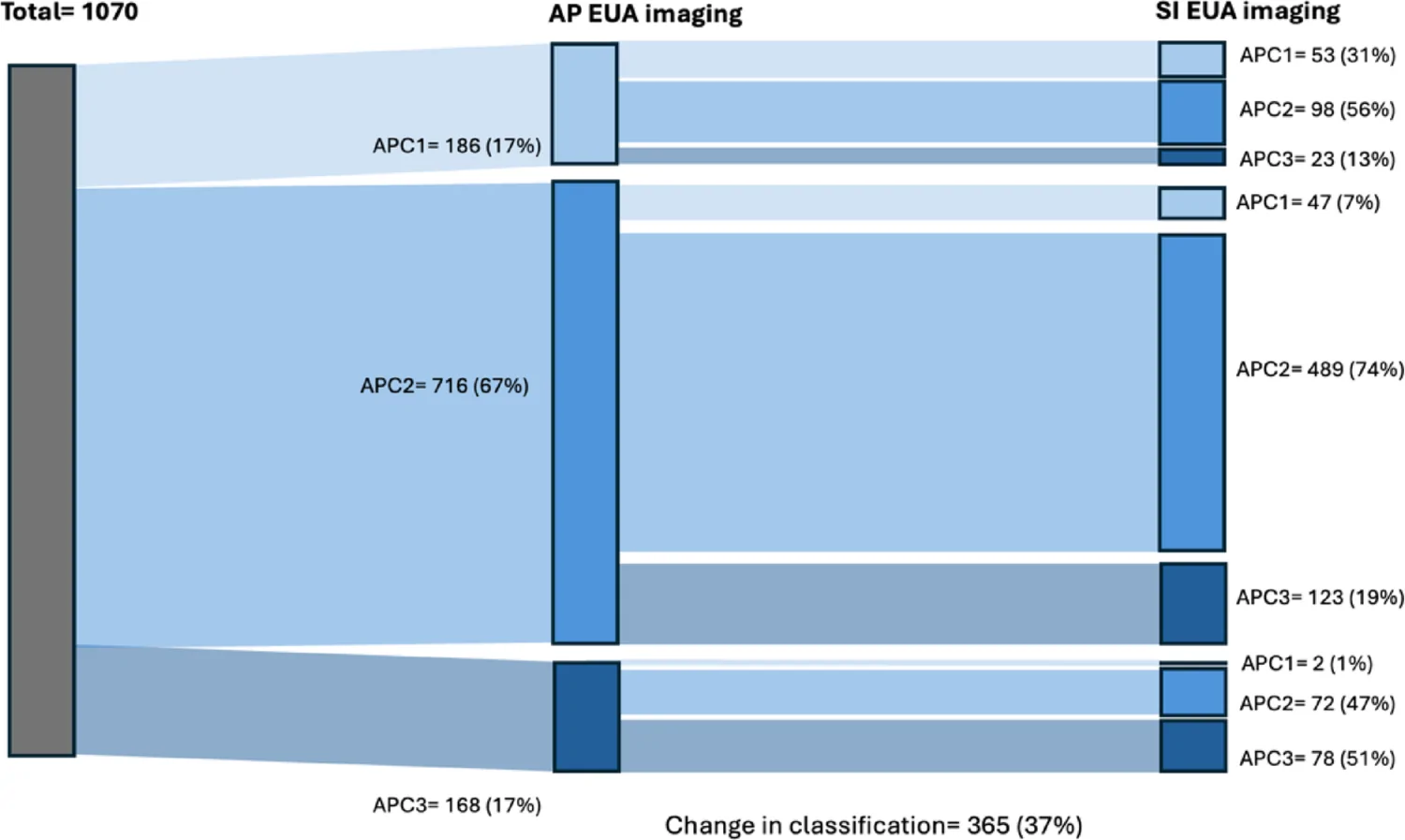
Changes in classification after the addition of SI EUA imaging

The classifications (APC1, APC2, or APC3) selected by the surgeons based on AP EUA imaging and SI EUA imaging, and the rates of change of classification between surveys are presented in Table 2. This data is presented graphically in online Appendix 2. Changes in classification were made in 365/992 (37%) cases. The specific changes made are visualized in Fig. 2. The flow diagram in Fig. 2 shows that, after the addition of SI EUA images, occult SI diastasis was identified in several patients, leading to reclassification from APC1 to APC2. In some cases, injuries were upgraded from APC2 to APC3, whereas others were deemed less severe and reclassified from APC3 to APC2. Overall, this resulted in a shift in classification patterns. The proportion of APC1 injuries decreased from 17 to 10%, APC2 remained unchanged at 67% in both surveys, and APC3 increased from 17% to 23% after the addition of SI EUA images.

**Table 2:** Classifications for each case based on static radiographs, CT-scans, and AP EUA images, compared with the addition of SI EUA images

Case*	Total	1	2	3	4	5	6	7	8	9	10
Survey 2: AP EUA images	(n = 1070)	(n = 106)	(n = 109)	(n = 106)	(n = 107)	(n = 108)	(n = 106)	(n = 106)	(n = 108)	(n = 108)	(n = 106)
APC1 n (%)	186 (17)	21 (20)	3 (3)	32 (30)	11 (10)	15 (14)	26 (25)	4 (4)	47 (44)	6 (6)	21 (20)
APC2 n (%)	716 (67)	75 (71)	66 (61)	45 (43)	82 (77)	75 (69)	75 (71)	79 (75)	52 (48)	88 (82)	79 (75)
APC3 n (%)	168 (17)	10 (9)	40 (37)	29 (27)	14 (13)	18 (17)	5 (5)	23 (22)	9 (8)	14 (13)	6 (6)
Survey 3: SI EUA images	(n = 992)	(n = 99)	(n = 99)	(n = 99)	(n = 101)	(n = 99)	(n = 99)	(n = 99)	(n = 99)	(n = 99)	(n = 99)
APC1 n (%)	103 (10)	8 (8)	2 (2)	21 (21)	3 (3)	2 (2)	9 (9)	2 (2)	31 (31)	1 (1)	24 (24)
APC2 n (%)	662 (67)	82 (83)	43 (43)	43 (43)	79 (78)	77 (78)	72 (73)	64 (65)	54 (55)	77 (78)	71 (71)
APC3 n (%)	227 (23)	9 (9)	54 (55)	35 (35)	19 (19)	20 (20)	18 (18)	33 (33)	14 (14)	21 (21)	4 (4)
Change of classification, n (%)	365 (37)	32 (33)	41 (41)	45 (46)	30 (30)	37 (37)	41 (42)	34 (35)	48 (49)	30 (30)	27 (28)

*Case numbers that had a pelvic binder on during static CT scans are underlined

### Changes in treatment decisions

**Fig. 3:**
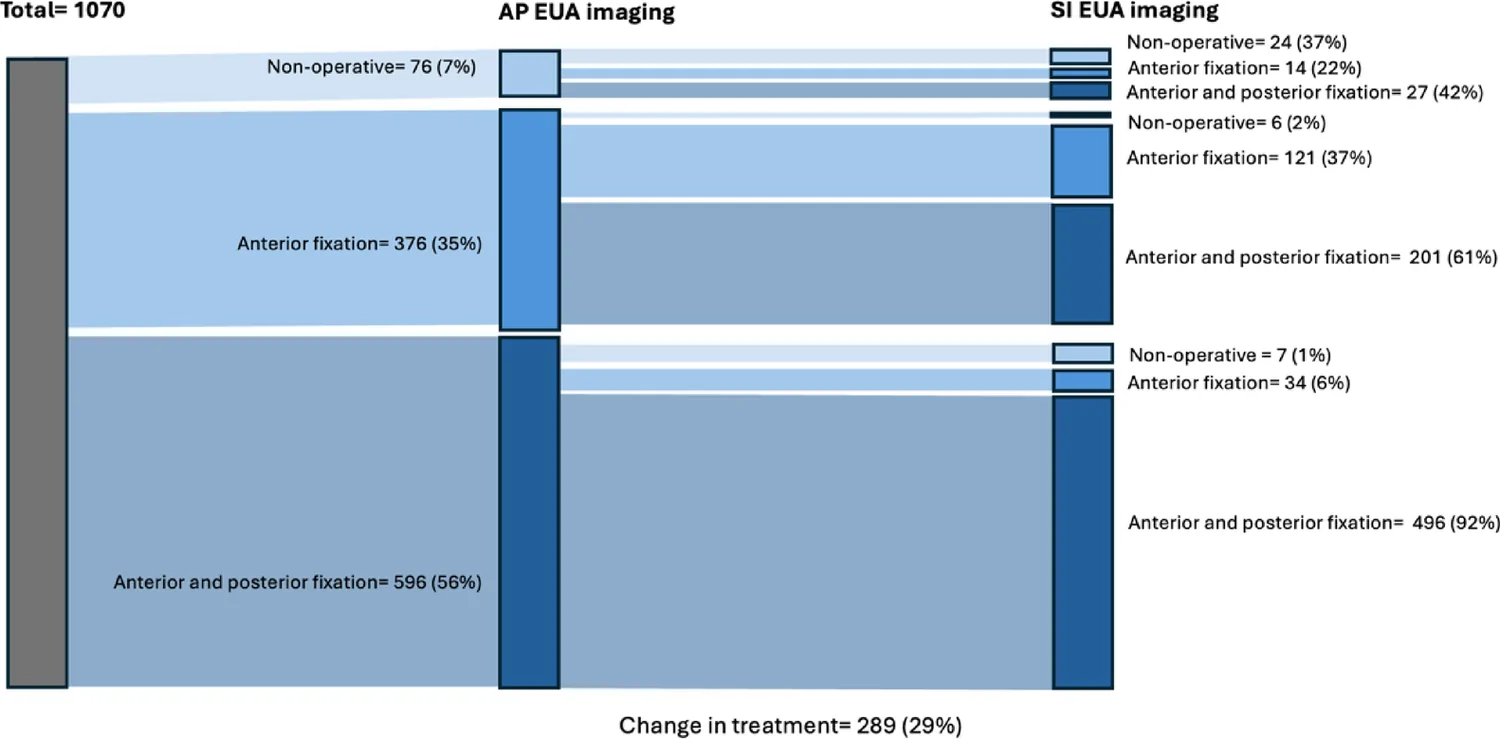
Changes in treatment decision after the addition of EUA imaging. The treatment choice “other” is not represented in this figure; refer to Table 3

The treatment decisions (non-operative, anterior only, or combined anterior and posterior fixation) selected by the surgeons based on AP EUA imaging and SI EUA imaging, and the rates of change in treatment decisions between surveys are presented in Table 3. This data is presented graphically in online Appendix 3. The total rate of change for treatment was 29% (289/992) in all cases. The specific changes made can be observed in Fig. 3. In all 10 cases, shifts towards a combined anterior and posterior fixation were observed, shifting from 56 to 74% after the addition of the SI EUA images.

**Table 3:** Treatment decisions for each case based on static radiographs, CT-scans, and AP EUA images, compared with the addition of SI EUA images

Case	Total	1	2	3	4	5	6	7	8	9	10
**Survey 2: AP EUA images**	(n = 1070)	(n = 106)	(n = 109)	(n = 106)	(n = 107)	(n = 108)	(n = 106)	(n = 106)	(n = 108)	(n = 108)	(n = 106)
Non-operative, n (%)	76 (7)	5 (5)	0 (0)	17 (16)	0	2 (2)	17 (16)	1 (1)	26 (24)	0	8 (8)
Anterior fixation only, n (%)	376 (35)	57 (54)	11 (10)	18 (17)	50 (47)	45 (42)	52 (49)	22 (21)	23 (21)	41 (38)	57 (54)
Anterior and posterior fixation, n (%)	596 (56)	43 (41)	96 (88)	59 (56)	57 (53)	60 (56)	36 (34)	82 (77)	55 (51)	67 (62)	41 (39)
Other, n (%)	22 (2)	1 (1)	2 (2)	12 (11)	0	1 (1)	1 (1)	1 (1)	4 (4)	0	0
Survey 3: SI EUA images	(n = 992)	(n = 99)	(n = 99)	(n = 99)	(n = 101)	(n = 99)	(n = 99)	(n = 99)	(n = 99)	(n = 99)	(n = 99)
Non-operative, n (%)	39 (4)	0	2 (2)	7 (7)	0	0	6 (6)	2 (2)	15 (15)	0	7 (7)
Anterior fixation only, n (%)	172 (17)	21 (21)	5 (5)	6 (6)	24 (24)	16 (16)	21 (21)	5 (5)	16 (16)	15 (15)	43 (43)
Anterior and posterior fixation, n (%)	736 (74)	75 (76)	89 (90)	67 (68)	75 (75)	83 (84)	69 (70)	88 (89)	59 (60)	83 (84)	48 (48)
Other, n (%)	43 (4)	3 (3)	3 (3)	19 (19)	0	0	3 (3)	4 (4)	9 (9)	1 (1)	1 (1)
Change of treatment, n (%)	289 (29)	39 (39)	11 (11)	27 (27)	29 (29)	33 (33)	43 (43)	20 (20)	24 (24)	30 (30)	33 (33)

*Case numbers that had a pelvic binder on during CT scans are underlined

### Right or left SI joint fixation

**Fig. 4:**
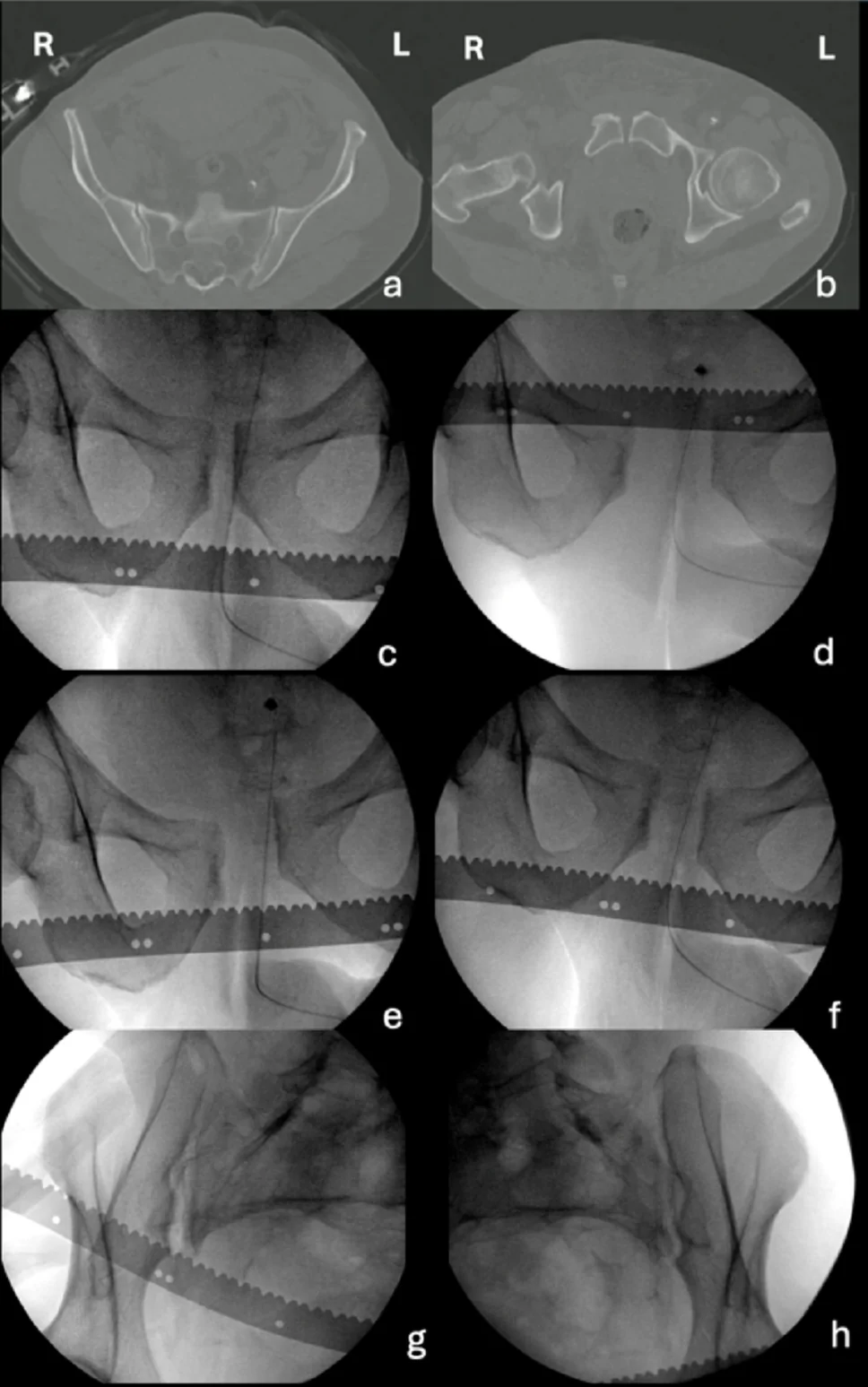
Case 1. This case demonstrates a significant change in treatment strategy after the addition of SI EUA images, shifting from predominantly anterior-only fixation (54%) to combined anterior and posterior fixation (76%). The SI EUA images helped localize the SI diastasis, leading to an increased rate of fixation of the right SI joint (from 31 to 77%). **a** CT scan showing the sacroiliac joint, **b** CT scan showing pubic symphysis, **c** AP EUA image in internal rotation stress (lateral compression), **d** AP EUA image in external rotation stress (frog position), **e** AP EUA image in push right leg/ pull left leg stress (rotational instability), **f** AP EUA image in push left leg/ pull right leg stress (rotational instability), **g** SI EUA image of right SI joint in external rotation stress (frog position)

Table 4 presents the frequency of right and left sided SI joint fixation selected in both surveys. This data is presented graphically in online Appendix 4. Based on the AP EUA images, the selected side of SI joint fixation in cases 1, 5, 6, and 9 was relatively evenly distributed between the right and left sides. After the addition of the SI EUA images, the distribution shifted toward a more clearly lateralized pattern, with surgeons more frequently selecting one side over the other. This suggests that direct visualization of the SI joints may help surgeons better identify the side of posterior pelvic ring instability when posterior fixation is considered. See Fig. 4 for an example of a case (case 1) in which the SI EUA images helped localize the SI joint instability and contributed to a change in the selected treatment strategy.

**Table 4:** Number of right and left SI joint fixations for each case based on static radiographs, CT-scans, and AP EUA images, and with the addition of SI EUA images

Case	1	2	3	4	5	6	7	8	9	10
Survey 2: AP EUA images	(n = 106)	(n = 109)	(n = 106)	(n = 107)	(n = 108)	(n = 106)	(n = 106)	(n = 108)	(n = 108)	(n = 106)
Right SI joint fixation, n (%)	33 (31)	16 (15)	4 (4)	54 (50)	40 (37)	31 (29)	11 (10)	58 (54)	60 (55)	37 (35)
Left SI joint fixation, n (%)	19 (18)	96 (88)	71 (67)	20 (17)	33 (31)	32 (30)	81 (76)	6 (5)	57 (52)	21 (20)
Survey 3: SI EUA images	(n = 99)	(n = 99)	(n = 99)	(n = 101)	(n = 99)	(n = 99)	(n = 99)	(n = 99)	(n = 99)	(n = 99)
Right SI joint fixation, n (%)	76 (77)	21 (21)	7 (7)	72 (73)	81 (82)	70 (71)	11 (11)	66 (67)	82 (83)	46 (46)
Left SI joint fixation, n (%)	7 (7)	91 (90)	85 (86)	45 (45)	27 (27)	55 (56)	91 (92)	32 (32)	58 (59)	16 (16)

## Discussion

This study found that the addition of direct visualization of the SI joints on pelvic EUA affected surgeons’ classification and treatment decisions for APC pelvic ring injuries [10]. The addition of SI EUA imaging led to a decrease in the proportion of APC1 classifications and an increase in APC3 classifications, while before and after the addition of SI EUA images, the majority of cases remained classified as APC2. More than one-third of cases were reclassified after applying the modified EUA protocol. These changes in classification were reflected in subsequent changes in treatment decisions. Across all cases, there was an increase in the use of combined anterior and posterior treatment approaches (56–74%). This suggests that direct visualization of the SI joints influences the surgeons’ impression of pelvic ring instability, prompting surgeons to choose a more extensive surgical strategy.

When the modified protocol was previously applied to a cohort in a Level I trauma center [10], 34% of patients demonstrated SI joint diastasis that would have been missed if posterior instability had been inferred solely from anterior pelvic displacement using the original EUA protocol with the standard cut-off values. This finding aligns with our results, in which 37% of surgeons reclassified the injury after incorporation of the modified SI EUA protocol. The importance of direct SI joint visualization with the modified EUA protocol is supported by multiple studies, indicating that anterior pelvic ring displacement alone is an unreliable indicator of posterior instability [10, 18, 22]. The 2.5 cm symphyseal diastasis cut-off value originated in 1980 and was based on cadaveric biomechanical observations [23]. This cut-off value was later adopted by Sagi et al. in the original EUA protocol [1]. However, Doro et al. recently demonstrated considerable variability in symphyseal widening (1.8–4.4 cm) at the point of anterior SI ligament failure, challenging the validity of a fixed threshold and reinforcing the need for direct SI joint assessment [18]. Similarly, MRI findings reported by Rojas showed that SI ligament injuries may be present even when anterior displacement remains below 2.5 cm [22]. These findings, along with the results of the present survey, support the addition of direct SI joint visualization during EUA as a critical component in assessing pelvic stability. This modification influences surgeons’ classification of APC injuries by revealing posterior instability that cannot be reliably predicted from anterior pelvic displacement alone.

According to the original EUA protocol [1], treatment recommendations are based on the degree of symphyseal diastasis and presumed posterior ligament injury. If the symphyseal diastasis is less than 2.5 cm, the sacroiliac ligaments are presumed intact (APC1), and non-operative treatment is recommended. If the diastasis exceeds 2.5 cm, indicating disruption of the anterior sacroiliac ligaments (APC2), anterior pelvic ring fixation is advised. In cases with more than 1 cm of rotational instability, posterior pelvic ring fixation is indicated [1]. This treatment algorithm was, however, not reflected by the treatment decisions made by this group of surgeons. In all cases, APC2 was the most frequently selected classification, while a combined anterior and posterior fixation approach was the most frequently chosen treatment strategy. Before the incorporation of the SI EUA images, many of these surgeons opted for anterior-only fixation. This indicates that direct observation of the SI joint diastasis motivates surgeons to stabilize the SI joint. The literature supports the surgeons’ treatment decisions, demonstrating that a combined fixation approach is beneficial in managing unstable APC injuries, as it enhances stability and reduces the risk of anterior plate failure [15, 17, 24, 25].

Furthermore, the modified EUA protocol provided information about which SI joint(s) should be stabilized. In several cases in our study, after the addition of SI EUA imaging, surgeons were able to differentiate instability between the right and left joints rather than relying on static imaging and anterior displacement only. While anterior-only EUA suggests the presence of SI joint diastasis based on anterior pelvic displacement, it cannot reliably indicate whether the instability involves the left, right, or both joints. The addition of direct SI visualization allowed surgeons to target fixation more accurately to the more unstable joint and choose a more tailored treatment. This aligns with previous findings showing that the modified EUA protocol enabled localization in almost half of the patient cohort [10].

Importantly, the clinical value of SI EUA imaging may extend beyond determining whether anterior-only fixation is sufficient. Some surgeons do not routinely perform anterior-only fixation for APC injuries because of concerns regarding fixation failure and therefore favour combined anterior and posterior fixation [17, 26, 27]. For these surgeons, direct SI joint visualization may still provide relevant information by helping to tailor the fixation strategy. Specifically, SI EUA imaging may influence the extent of posterior fixation, including the number of posterior screws used, and help determine whether unilateral or bilateral SI joint fixation is required. Therefore, even when posterior fixation is already being considered, SI EUA imaging can provide additional information that may guide a more targeted and individualized treatment approach.

Some limitations of this study should be acknowledged. First, the survey relied on surgeons’ interpretations of clinical case scenarios, which may not fully capture the complexity of intraoperative decision-making, but still closely represent clinical practice. Furthermore, the study introduced a new EUA imaging technique without additional guidance, leaving surgeons to interpret the images on their own, which allows variability in how the images were utilized. On the other hand, this approach provides a global indication of how the additional images influence surgeons’ impressions of the injuries. In addition, the study did not assess the consistency of surgeons’ decision-making over time, as repeated evaluations of the same cases, with the same imaging, were not performed. Finally, while changes in classification and treatment were documented, the study did not assess whether these changes would translate into improved patient outcomes, such as successful fixation or functional recovery. Despite these limitations, the survey offers valuable insight into how the addition of SI EUA imaging affects surgical interpretation and decision-making for APC pelvic ring injuries.

## Conclusions

The addition of SI EUA images according to the modified protocol alters the surgeon’s perception of the level of instability of the pelvic ring and consequently also the treatment decisions. The overall rate of change of classification was 37%, with an overall decrease in APC1 and an increase in APC3. Moreover, the rate of change for treatment decisions was 29%. There was an increase in the combined anterior and posterior treatment approach in all ten cases. The addition of the SI EUA images also enabled more precise localization of the injury, allowing surgeons to determine which sacroiliac joint required stabilization. Therefore, SI EUA images are a valuable addition to the EUA technique, as they help further identify occult posterior pelvic ring instability and inform treatment decisions.

## Supplementary Information

Below is the link to the electronic supplementary material.

Supplementary Material 1

Supplementary Material 2

Supplementary Material 3

Supplementary Material 4

## Data Availability

No datasets were generated or analysed during the current study.
